# Artificial Crowns

**Published:** 1894-12

**Authors:** J. H. Crossland

**Affiliations:** MONTGOMERY, ALA.


ARTICLE V.

### ARTIFICIAL CROWNS.

DR. J. H. CROSSLAND, MONTGOMERY, ALA.

Mine is a band crown, the band placed in a groove cut in the end of the root, as near as practical to the circumference. Grind the root a little beneath the gum margin all around, as you would for an ordinary porcelain crown. With a fine drill (preferably one provided with a gage) drill a series of holes, as near as practicable to the circumference, about a thirty-second of an inch deep, and as close together as possible. Cut these together, and fit into the groove thus formed a band of gold 28 or 30 gage, 24k., and sufficiently wide to reach bottom of groove and project a trifle beyond surface of root end. Grind this to level of root. From pure gold, 34 gage, cut a cap to proximately fit root end. Place in position and burnish to edge of band, remove all and place band in position on cap, which will be plainly indicated by the fact that it will fit only in one position. Solder with 20k. solder, using as little as possible. Replace and burnish thoroughly to root. Punch and place platinum post in position, and secure with hard wax. Remove, invest, and solder. Trim accurately to outlines of root end. Place in position again. Back plate tooth with gold, and proceed as with ordinary porcelain faced crown.

In backing, grind plate tooth to fit cap, bevel from pins to cutting edge, reducing it almost to a knife edge. Grind this edge away, leaving the end square or beveled a little toward labial surface. Cut 34 gage, 24k. gold backing, slightly longer than tooth, punch, place in position and secure by spreading pins. Burnish thoroughly to tooth from tip to tip, then over cutting edge to square end, or beveled surface on labial aspect. Trim accurately, allowing no gold to extend beyond square end or beveled surface on front, as it would cause your tooth to crack on soldering, Cut another backing long enough to burnish over cutting edge, but not further than the slope of the tooth toward gingival end. Punch in this a series of small holes to insure a perfect union in soldering. Straighten pins, place in position, secure as you did in backing number one, burnish over end of tooth, and trim accurately. This is to thicken gold on cutting edge, and to prevent fusing of outer backing. Grind gold on gingival end of tooth to fit cap. Place all in position and unite with wax, being careful that joint between gold on end of tooth and cap is covered with wax, so that none of your investing material can get in to prevent a thorough soldering.

Extend wax over all gold exposed on cutting, so that it will be exposed when wax is melted out of investment. Invest in the usual way. Heat very hot before beginning to solder. Use very little solder to unite cap and backing, and continue heat for a moment before placing more solder, which causes it to flow thoroughly into the minute crevice between cap and backing, making a beautiful joint. Use 20k. solder, as a little of it may be exposed at cutting end of tooth. If desired, an extensive tipping can be made by placing pieces of gold plate at this point, in the investment, or by using a number of backings and burnishings over end.

Exposure of gold, presenting the appearance of fillings can be made in the same way. The mechanical advantage of the gold extension at this point is of course apparent, and the slight esthetic objection can be reduced to a minimum, as by prop or beveling a small amount of gold can be made to serve the purpose. I have used this principle in bridge-work also, and am much pleased with it. If it were generally used, we would hear little discussion as to methods of constructing bridges so that teeth can be put on without removing piece. It is less bulky and more shapely than the old ferrule crown; exposes no gold at gum margin; allows a much more favorable and accurate adjustment with regard to position of other teeth; can be used where strikingly convergent or divergent roots are to severe as abutments for bridge; is especially adapted to conically-shaped roots; causes less pain in adjustment, and will not irritate pericemental membranes. It is extremely cleanly.

To those having fondness for the ceramic art, I suggest that this form of attachment can be used to advantage by making the band and cap of platinum and baking in porcelain backing, producing a highly esthetic crown; possessing to some extent the same mechanical advantages as if backed with gold, etc.

A crown provided with four extra posts as a substitute for inside band can be used to advantage in some cases, backed either with gold or porcelain.—*Busy Dentist.*

